# A transit passenger with unilateral leg pain

**DOI:** 10.1002/emp2.13221

**Published:** 2024-09-23

**Authors:** Ghazala Faheem, Muhammad Abd Ur Rehman, Muhammad Junaid Iqbal, Tahir Shahzad

**Affiliations:** ^1^ Department of Emergency Medicine Hamad Medical Corporation Doha Qatar

## 1 PATIENT PRESENTATION

**FIGURE 1:**
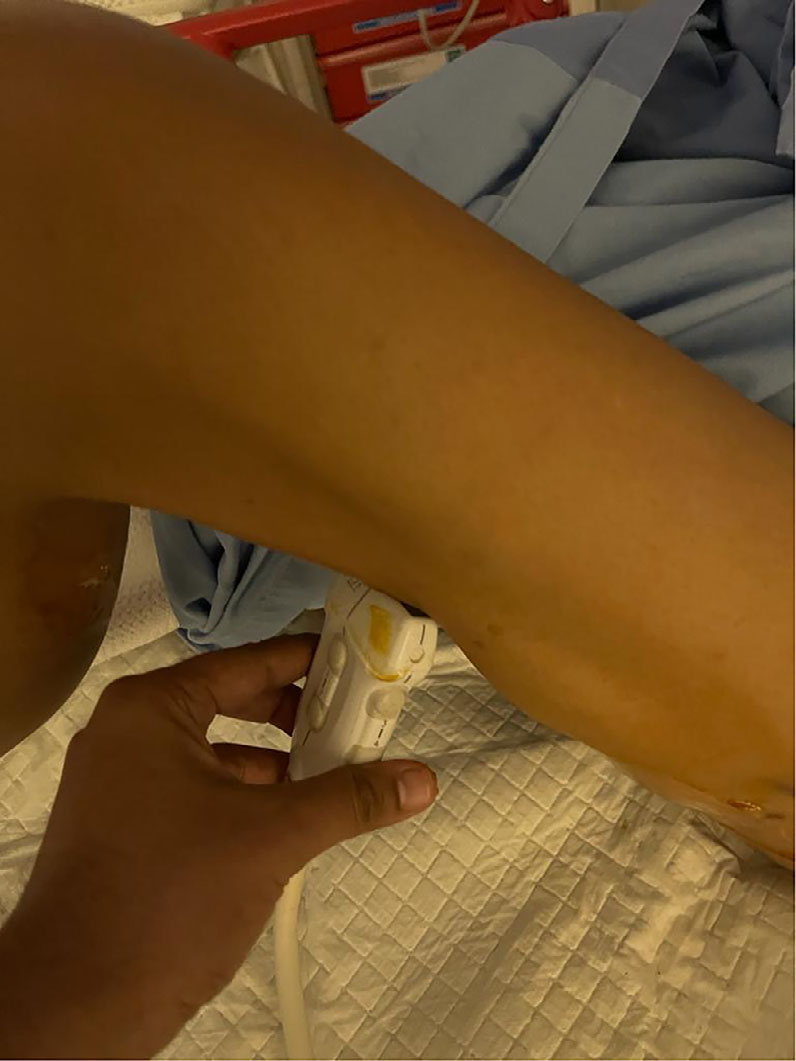
Showing position of the high‐frequency linear array transducer for thigh ultrasound.

A 57‐year‐old male transit passenger from an 8‐hour flight presented to the emergency department with severe left posterior thigh pain. He had experienced a traumatic event a few weeks prior, resulting in fractures of the left hip and left lower ribs. Upon examination, he exhibited severe tenderness, warmth, and fluctuation in the left posterior thigh with minimal swelling. He had a low‐grade fever (37.8°C), was markedly tachycardic (heart rate: 125 beats/min), and appeared clinically dehydrated. Blood investigations revealed raised inflammatory markers, elevated creatinine, and high lactate levels (Table 1). Given his clinical presentation and recent surgery combined with the long flight, initial differential diagnoses included deep venous thrombosis (DVT) and sepsis. Point‐of‐care ultrasound (POCUS) ruled out DVT but revealed subcutaneous edema with fluid collection in the hamstring muscles (Figure 1, Video 1).

**TABLE 1:** Lab investigations.

Blood investigations	Patient's values	Normal range
White blood cell (× 10^3^/µL)	22.7	4.0–10.0
C‐reactive protein (mg/L)	340	0.0–5.0
d‐Dimer (mg/L FEU)	0.33	0.00–0.49
Creatinine (µmol/L)	151	62–106
Lactic acid (mmol/L)	5.5	0.5–2.2
pH	7.11	7.35–7.45
PCO_2_ (mmHg)	38	21–69
PO_2_ (mmHg)	27	25–40
HCO_3_ (mmol/L)	20	23–29

**VIDEO 1:** Point‐of‐care ultrasound (POCUS) showing subcutaneous edema with fluid collection in muscles

## 2 DIAGNOSIS

### 2.1 Pyomyositis with sepsis

Based on laboratory investigations and bedside ultrasound findings, a diagnosis of pyomyositis with sepsis was made. The patient was promptly administered broad‐spectrum antibiotics and surgical consultation was obtained, leading to the incision and drainage of the thigh abscess, with approximately 100 mL of pus drained in the operation room. The patient showed significant clinical improvement and was discharged in stable condition after 3 days. Pyomyositis, a suppurative infection of skeletal muscle often affecting the proximal lower extremities, can lead to abscess formation.^1^, ^2^ It progresses through three stages. In the first stage, the muscle becomes inflamed and painful, with a “woody” texture but no abscess or erythema, and may show mild leukocytosis. The second stage involves severe pain, swelling, fever, and the formation of a muscle abscess, typically lasting 1–3 weeks. The third stage features systemic toxicity, septicemia, multifocal abscesses, and shock.^3^ POCUS enables rapid diagnosis and facilitates prompt surgical consultation following the immediate administration of broad‐spectrum antibiotics in the emergency dt.^4^ Ultrasound is cost effective and is highly effective at distinguishing fluid from solid tissue. In musculoskeletal infections, its main use is diagnosing and locating fluid in soft tissues or joints.^5^ Early‐stage disease, marked by firm and tender muscle, can be effectively treated with beta‐lactamase‐resistant penicillin, leading to recovery within 2–3 weeks. Later stages or unresolved early stages often produce pus on aspiration and require incision, drainage, and antibiotics.^3^
